# Innovation and Precision Medicine Applied to a Medullary Thyroid Cancer: A Clinical Case

**DOI:** 10.7759/cureus.40107

**Published:** 2023-06-07

**Authors:** Helena Guedes, Inês Leão, Adriana Soares, Raquel Basto, Ana Joaquim

**Affiliations:** 1 Oncology Department, Centro Hospitalar Vila Nova de Gaia/Espinho, Vila Nova de Gaia, PRT

**Keywords:** targeted therapy, ret, clinical trials, precision medicine, medullary thyroid cancer

## Abstract

Medullary thyroid carcinoma (MTC) is a rare type of neuroendocrine tumor, accounting for 3%-4% of all thyroid cancers. Seventy-five percent are sporadic, of which 60% have pathogenic REarranged during Transfection (RET) somatic mutations. The sporadic RET-mutated MTC poses novel challenges for targeted treatment. The authors present a case of a 60-year-old male diagnosed in 2018 with MTC who underwent total thyroidectomy with sternotomy and bilateral cervical lymph node dissection - pT3N1b R1 L1 V1 Pn0 cM1 (hepatic and lung metastasis). According to the decisions made by the multidisciplinary tumor board, the patient received multiple palliative systemic treatments. Despite an initial response, vandetanib was accompanied by grade 3 high blood pressure and progression after 14 months of treatment. The patient also received cabozantinib, which led to an initial response, but with grade 3 hypertension and skin toxicity. The patient progressed, including symptomatic bone metastasis, after 15 months of treatment. Following the next sequencing genome result, which showed a somatic mutation in the RET M918T gene, the patient was treated with selpercatinib, a highly selective and potent RET inhibitor. The treatment led to clinical and radiological responses without significant toxicities. The objective of this case report is to highlight the impact of innovative treatment and precision medicine on the management of cancer patients, which not only has a direct effect on their survival but also on their quality of life.

## Introduction

Medullary thyroid cancer (MTC) is a rare type of neuroendocrine tumor, accounting for approximately 3%-4% of all thyroid cancers [1]. Approximately 20%-25% of MTC cases are hereditary and linked to multiple endocrine neoplasia type 2 (MEN2) syndrome, while most cases (75%) are sporadic and typically diagnosed between the ages of 45 and 55. Screening and surveillance for hereditary MTC and prophylactic thyroidectomy are associated with improved outcomes in patients with germline RET proto-oncogene mutations [2]. About 60% of these sporadic cases are found to have somatic RET mutations [3-5].

At the time of diagnosis, around 10% of cases are diagnosed with distant metastases, and approximately 15% develop metastatic disease during follow-up, which is linked to a poorer prognosis. MTC-RET-mutated is associated with a more aggressive form of the disease [6]. For patients with advanced metastatic MTC, cabozantinib and vandetanib, nonselective RET inhibitors, were the first targeted systemic therapies approved regardless of RET status, although subgroup analyses suggest improved response in patients with RET mutations or M918T mutations specifically [7,8]. These drugs have a similar toxicity profile [4,7,9].

In 2020, a phase 1-2 trial involving 143 patients demonstrated the long-lasting effectiveness of selpercatinib, a highly selective and potent RET inhibitor, in treating patients with RET-mutated MTC, independently of previous treatment with vandetanib or cabozantinib [1]. The clinical trial demonstrated manageable adverse effects, with the predominant grade 3 or 4 events (according to the Common Terminology Criteria for Adverse Events, version 5) being hypertension (in 21% of patients), increased serum alanine aminotransferase levels (in 11% of patients), increased serum aspartate aminotransferase levels (in 9% of patients), hyponatremia (in 8% of patients), and diarrhea (in 6% of patients) [4]. This trial led to the approval of the drug by the Food and Drug Administration (FDA) in 2020 and subsequently by the European Medicines Agency (EMA).

Selpercatinib monotherapy is now indicated for the treatment of advanced RET-mutant MTC in adults [10]. The authors present a case of a 60-year-old male diagnosed with cervical lymph nodes, liver, and lung metastases of medullary thyroid carcinoma in 2018 who underwent surgery (R1) with two previous lines of treatment with vandetanib and cabozantinib. A next-generation sequencing analysis performed at the time of disease progression in 2021 revealed the presence of the RET M918T somatic mutation, and a request for selpercatinib was made. The goal of this case report is to highlight the impact of precision medicine on the management of cancer patients, which not only has a direct effect on their survival but also their quality of life.

## Case presentation

The patient is a 60-year-old black male who has a medical history of hypercholesterolemia, arterial hypertension grade 1, hepatitis B, and a former smoking habit of 90 pack years. The patient is currently on regular medication, which includes bisoprolol 2.5 mg once a day and simvastatin 20 mg once a day. There is no family history of cancer.

**Figure 1:**
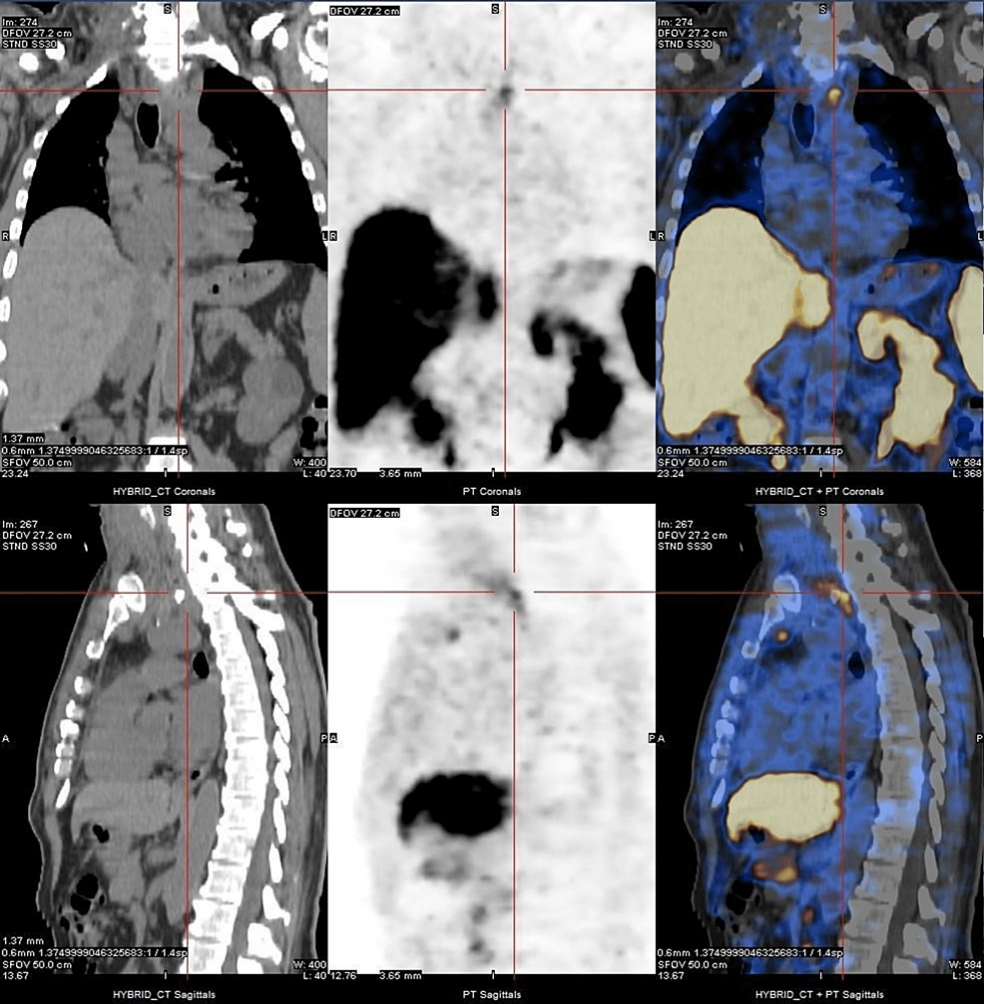
PET CT at diagnosis

In 2018, the patient presented to the attending physician with bilateral nodules located in the anterior cervical region that had been increasing gradually over three years, accompanied by anorexia and weight loss (20% of the weight in six months). No other symptoms or signs. Eastern Cooperative Oncology Group (ECOG)-Performance Status 1. In 08/2018, the imaging study (cervical and thoracic computed tomography (CT) and positron emission tomography (PET)) showed a large, well-delimited, homogeneous expansive mass with a diameter of 47x64mm, located in the posterior mediastinum, left paratracheal area, with a significant mass effect on the trachea and esophagus. The patient also had multiple nodular formations in the cervicothoracic gorge with dimensions of 30mm, retro thyroid, and retroclavicular, bilaterally, as well as suspected pulmonary and hepatic metastasis. A core biopsy of the left cervical lymph node confirmed the diagnosis of cervical metastasis of medullary thyroid carcinoma with hepatic and pulmonary metastases (Figure 1).

After discussion in a multidisciplinary discussion team (MDT), the patient underwent a total thyroidectomy with sternotomy and bilateral cervical lymph node dissection in September 2018 (pT3N1b R1 L1 V1 Pn0). The patient had a complication in the postoperative period, an accidental extubation, and was unable to reintubate due to edema of the oropharyngolaryngeal structures with desaturation, requiring an emerging tracheostomy. Postoperative imaging studies, two months after surgery (PET-CT), showed the persistence of disease in the upper mediastinum, laterocervical ganglionic metastases, right and mediastinal, lung, and liver metastases. The MDT proposed vandetanib (300 mg once a day, orally), which the patient started on 16/01/2019. No germline RET mutations were identified. The patient had hypertension grade 3, fatigue grade 2, and grade 3 acneiform skin rash (CTACE v.5) [11] on the face, chest, and back, requiring drug withdrawal on 30/07/2019 (six months of treatment). He resumed the drug on 08/2019, after dermatological evaluation, with maculopapular rash grade 1 and dose reduction (200 mg/day). After dose reduction, the patient had control of side effects and showed partial response until 03/2020. The patient had ECOG performance status 2, and the imaging evaluation (PET-CT) showed progressive disease in the lungs and liver. On 28/04/2020 he resumed treatment with a dose reduction (40 mg once a day), but due to grade 2 diarrhea (CTCAE v.5), he suspended treatment on 08/07 and restarted on 14/08/2020 with a dose reduction. According to the Naranjo Algorithm, hypertension and diarrhea are definite drug-associated adverse events; rash is probable; and fatigue is a possible adverse event [12].

After discussion in the MDT, the patient started cabozantinib (60 mg once a day, orally) on 17/03/2020. A genomic panel was also requested. The patient experienced G3 acneiform rash, G2 pruritus, and G2 diarrhea (CTCAE v.5) during treatment. He was evaluated by immunoallergology and dermatology, and it was decided to suspend treatment for 12 days (15/04 to 27/04/20202) and adjust support medication.

**Figure 2:**
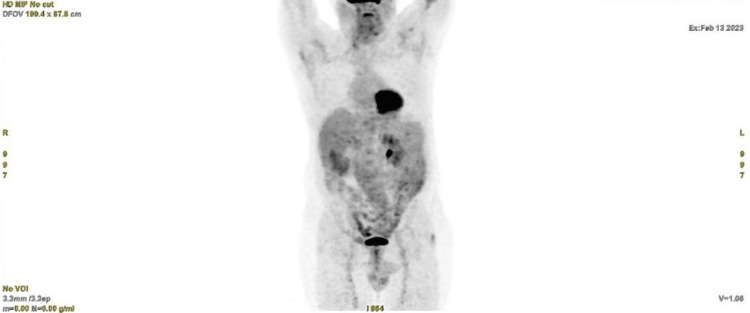
PET CT at 18 months of treatment with selpercatinib

The disease remained stable until 06/2021, when a PET-CT scan showed progression of the disease, with the progression of liver lesions and the appearance of neoplastic conglomerates, again, interaortocaval metastases and symptomatic bone metastases, prompting the initiation of analgesic therapy with opioids. The patient was enrolled in the EORTC SPECTA-Arcagen program (NCT02834884). A molecular profile was performed on formalin-fixed paraffin-embedded (FFPE) using the Foundation One CDx. The next-generation sequencing (NGS) results revealed a somatic mutation in the RET M918T gene with a VAF of 24.91%. The MDT proposed selpercatinib (160 mg twice a day, orally), which the patient started on 10/08/2021. During the treatment, the patient needed to adjust support medication due to G2 diarrhea. Currently, treatment with selpercatinib is ongoing, on the 18th month, with a resolution of the bone pain and partial response as the best response (Figure 2).

Importantly, the patient presents ECOG performance status 1 and has returned to professional activity for the first time since he started the treatments.

## Discussion

Historically, advanced MTC was treated with surgery or radiation therapy, which had varying success rates and significant associated morbidity. However, recent advances in targeted therapies have led to important changes in standard treatment protocols in the setting of advanced-stage disease and improved long-term outcomes for patients with locally advanced, metastatic, or recurrent MTC [2]. Multikinase inhibitors such as cabozantinib, lenvatinib, and vandetanib have shown activity in RET-dependent malignancies, while selective RET inhibitors such as selpercatinib and pralsetinib have been developed to achieve higher potency anti-tumor effects with less toxicity, resulting in improved side effect profiles compared to multikinase inhibitors, likely due to less activity against VEGFR2 [2,11].

This case presents a patient diagnosed with MTC in 2018 who underwent R1 surgery and was subsequently proposed for several lines of targeted therapy, including vandetanib and cabozantinib. While the patient experienced disease progression after the first two lines of treatment, the identification of a somatic mutation in the RET M918T, based on the results of the LIBRETTO-01 trial at the time, led to the patient starting a third line of treatment with selpercatinib (160mg orally, twice a day). Selpercatinib was evaluated in phase I/II single-arm, multicenter, open-label, multicohort clinical trials in patients with advanced solid tumors harboring RET fusions and mutations. The efficacy results for patients with metastatic RET-mutant MTC showed positive responses in patients with M918T mutations and other mutations [2]. The patient benefited from all the previous lines of treatment with targeted therapies, which had a significant global impact on overall survival.

Regarding adverse events (AE), the most relevant were dry mouth (39%), hypertension (30%, 12% grade 3), diarrhea (17%, 3% grade 3), and fatigue (25%, 1% grade 3) [4]. As described in the literature, in this clinical case, the most frequent side effect was diarrhea, which was self-limited and managed with supportive measures, allowing the patient to maintain his daily activities and quality of life.

Although the patient experienced adverse events, most of these side effects were mild to moderate in severity and managed with supportive measures. It was crucial to monitor the patient's side effects to ensure that the patient could return to work and maintain daily care activities.

There are other promising selective RET inhibitors currently under investigation in the management of MTC, which may lead to additional treatment options and overcome resistance to other tyrosine kinase inhibitors (TKIs). Based on these data showing good efficacy and better side effect profiles, patients with symptomatic or progressive metastatic MTC should be treated with selpercatinib or pralsetinib [2].

The presented case demonstrates the example of a long-term survivor diagnosed with a rare neoplasm, highlighting the importance of precision medicine. Identifying RET mutations in MTC and developing RET inhibitors with increasing selectivity have expanded the therapeutic options for effectively controlling tumor growth in progressive MTC that has metastasized to distant sites. However, preventing the development of resistance to these selective RET inhibitors poses a new challenge. Fortunately, the growing understanding of the molecular mechanisms underlying such resistance holds promise for further improving patient outcomes.

## Conclusions

We have described a case of sustained response in a patient with advanced metastatic sporadic MTC RET-mutated who was treated with selpercatinib after two previous lines of treatment of TKI. These systemic targeted therapies hold great promise for improving the management of patients with locally advanced or metastatic disease, with an important impact on their daily life activities. Ongoing research is being conducted to determine the optimal use of these new drugs to enhance the care of patients with MTC.
